# Impact of clinical and health services research projects on decision-making: a qualitative study

**DOI:** 10.1186/1478-4505-11-15

**Published:** 2013-05-10

**Authors:** Maite Solans-Domènech, Paula Adam, Imma Guillamón, Gaietà Permanyer-Miralda, Joan MV Pons, Joan Escarrabill

**Affiliations:** 1 Agency for Health Quality and Assessment of Catalonia (AQuAS), Roc Boronat 81-95, Barcelona, 08020, Spain; 2 CIBER Epidemiologia y Salud Pública, Roc Boronat 81-95, Barcelona, 08020, Spain; 3 Epidemiology Unit, Cardiology Service, Vall d’Hebron Hospital, Passeig de la Vall d'Hebron, 119-129, Barcelona, 08035, Spain; 4 Chronic Care Program Hospital Clínic, Villaroel 170, Barcelona, 08036, Spain; 5 Network on Health Services Research in Chronic Diseases (Red de Investigación en Servicios de Salud en Enfermedades Crónicas) (REDISSEC), Villaroel 170, Barcelona, 08036, Spain

**Keywords:** Informed decision-making, Qualitative research, Research impact, Respiratory diseases, Payback model

## Abstract

**Background:**

This article reports on the impact assessment experience of a funding program of non-commercial clinical and health services research. The aim was to assess the level of implementation of results from a subgroup of research projects (on respiratory diseases), and to detect barriers (or facilitators) in the translation of new knowledge to informed decision-making.

**Methods:**

A qualitative study was performed. The sample consisted of six projects on respiratory diseases funded by the *Agency for Health Quality and Assessment of Catalonia* between 1996 and 2004. Semi-structured interviews to key informants including researchers and healthcare decision-makers were carried out. Interviews were recorded, transcribed verbatim and analysed on an individual (key informant) and group (project) basis. In addition, the differences between achieved and expected impacts were described.

**Results:**

Twenty-three semi-structured interviews were conducted. Most participants indicated changes in health services or clinical practice had resulted from research. The channels used to transfer new knowledge were mainly conventional ones, but also in less explicit ways, such as with the involvement of local scientific societies, or via debates and discussions with colleagues and local leaders. The barriers and facilitators identified were mostly organizational (in research management, and clinical and healthcare practice), although there were also some related to the nature of the research as well as personal factors. Both the expected and achieved impacts enabled the identification of the gaps between what is expected and what is truly achieved.

**Conclusions:**

In this study and according to key informants, the impact of these research projects on decision-making can be direct (the application of a finding or innovation) or indirect, contributing to a more complex change in clinical practice and healthcare organization, both having other contextual factors. The channels used to transfer this new knowledge to clinical practice are complex. Local scientific societies and the relationships between researchers and decision-makers can play a very important role. Specifically, the relationships between managers and research teams and the mutual knowledge of their activity have shown to be effective in applying research funding to practice and decision-making. Finally the facilitating factors and barriers identified by the respondents are closely related to the idiosyncrasy of the human relations between the different stakeholders involved.

## Background

The importance of understanding research impact and how research findings are translated into practice or put into action is widely accepted [1]. However, the knowledge translation from research into practice may not be achieving an optimal exploitation [2-4]. This process may have low intensity and be unpredictable [5] and often not dedicated to informing decision-making [6]. The present economic climate and increased competition for public funds makes this even more critical. In health sciences, the term “research impact” refers to any type of output or outcome of research activities which can be considered a “positive return or payback” for the scientific community, health systems, patients, and society in general [7,8]. Several evaluative frameworks and methods to capture the returns of health research investment have been proposed [9].

One of these models, the Return of Investment (ROI), was developed by the Canadian Academy of Health Sciences (CAHS) [10]. The CAHS-ROI conceptual framework derives from an adaptation of the payback model from the Brunel University [11,12]. The framework allows the impact of different types of health research (basic biomedical, applied clinical, health services and systems, and public population health) to be traced in five main categories: advancing knowledge, research capacity-building, informing decision-making, health impact, and broad economic and social impact [13]. Each category includes a set of impact indicators with accepted standards of validity and feasibility [14].

The final impact of research, however, will be influenced by the quality of the research itself and by the extent to which the knowledge obtained is made available to those in a position to use it. Therefore, knowledge transfer, its adoption (adaption), and its application through changes in behaviour or decision-making creates different interfaces among different subjects, mainly between research producers and knowledge users. The field of implementation science deals with this transfer which goes beyond the simple dissemination of knowledge [15]. In the translation of biomedical research findings it is important to differentiate between two main types of transfer: T1 that deals with the application of new understandings of disease mechanisms gained in the laboratory into new methods for diagnosis, therapy, and prevention and their initial testing in humans and, equally important, T2 referring to the translation of results from clinical studies into everyday clinical practice and health decision-making [16].

In the CAHS-ROI model, as in its payback predecessor, the topic of decision-making implicitly includes a whole array of considerations regarding the model’s interfaces, the transfer or exchange of knowledge, and the interrelationship between scientific researchers and decision-making communities [17]. This is the most difficult part to analyse since, in real life, the interrelationship is not linear or unidirectional.

This article is based on the assessment experience of a funding program of non-commercial clinical and health services research promoted in Spain by the *Agency for Health Quality and Assessment of Catalonia* (hereinafter the Agency) [18]. The aim of this extramural research program is not only to fill knowledge gaps but also to aid decision-makers in the healthcare sector, or, to use the terminology of the different logic models of impact assessment, to have an impact on informed decision-making processes.

The specific aim of the study is to assess the level of implementation of results from a subgroup of research projects (dealing with respiratory diseases), based on the opinion of researchers and a different typology of healthcare decision-makers as well as to identify the barriers or facilitators of translating new knowledge into informed decision-making. The study also addresses the multidimensional issue commented by Weiss [19] on the decision-making process that is differentiating awareness from implementation and the similarly important aspect of who takes the role of decision-maker or person in charge of translating research outputs into daily practice (implementation).

## Methods

A qualitative study with interviews was carried out. It is an adequate method to explore and understand the perceptions of key informants about a particular social phenomenon.

### Setting and sample

The sample consisted of a subgroup of multicentre projects on respiratory diseases which were funded between the 1996 and 2004 by the Agency calls. The topic was chosen for convenience and its selection was performed based on pragmatism, feasibility, and accessibility. The sample had to allow for the analysis of a manageable number of projects. The selection of projects needed to include available information and readily accessible key informants. Only projects completed at the time the study was performed were included.

### Data collection procedures

Semi-structured interviews to key informants and included researchers and healthcare decision-makers were carried out. Clinical group leaders and organizational or administrative managers in healthcare institutions were considered to be healthcare decision-makers.

Principal investigators (PIs) of the selected projects were identified as eligible key respondents. We therefore identified participants based on their significance. With regard to healthcare decision-makers, heads of hospital respiratory services, medical directors and general managers of centres who participated in the funded research were identified as potential ‘users’ of project results. The search for the healthcare decision-makers was made over the research period of the funded projects and up until the present. The eligible decision-makers interviewed needed to be aware of the selected projects, so a screening questionnaire was used to assess whether they were aware of these. Following this, snowballing techniques were used to identify researchers and decision-makers who could provide the most information regarding the utilisation of the research. Those who accepted to participate were contacted to arrange an interview.

The number of participants differed depending on the number of relevant researchers, decision-makers, and institutions participating in each project.

The interview guide was developed by our team, for each group of key informants (researchers and decision-makers), based on the objectives of the study. The interviewer received prior information and documentation about the funded projects and their impact assessment following the framework pattern. The interviews, which on average lasted between 15 and 30 minutes, were conducted in a place chosen by the interviewees. Interviews were carried out between April and November 2010. The participants in the study were previously informed of the study objectives and the characteristics of the interview procedure. They were asked to give their consent for the interviews to be recorded and were ensured of confidentiality. In order to maintain anonymity, every informant interviewed was given a code number so as to preclude identify them.

The interviews with PIs were semi-structured face-to-face. They included aspects related to: a) the expected and use of research results and that achieved in decision-making in different sectors: clinical, management, research, political, etc. (the question addressing the expected use of results was formulated to identify the preconceived notion that researchers had on what the impact of the research project ‘should be’; in terms of uses achieved, the question implicitly attempted to encourage the researcher to reflect on ‘what really happened’, or what the achieved impacts were); b) the dissemination of research results; and c) barriers and facilitators in the translation of research, among others.

For healthcare decision-makers, semi-structured interviews were carried out by phone, and the issues covered included: a) the influence of project results on healthcare management; b) the implementation of changes; and c) possible barriers or facilitators for their acceptance.

### Data management and analysis

**Figure 1:**
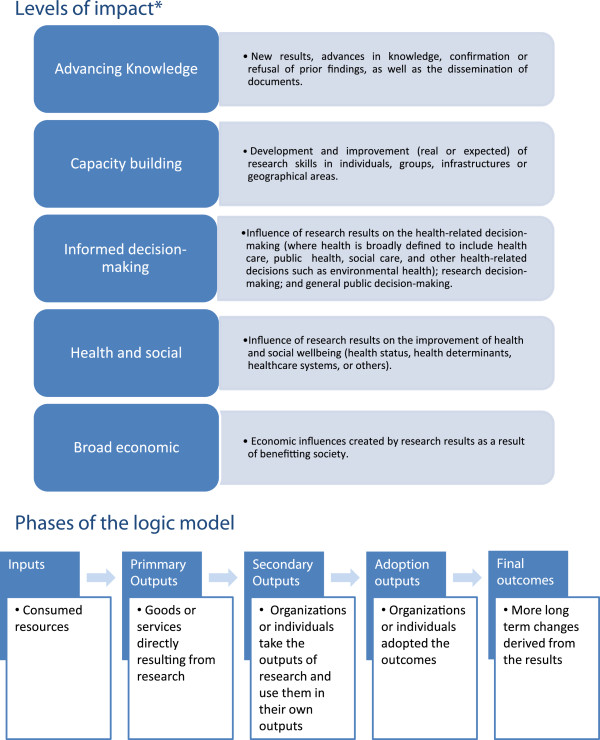
**Levels of impact and phases of the Canadian Academy of Health Sciences (CAHS-ROI) model.** *Definitions adapted from Panel on the return on investments in health research [14]

Interviews were recorded and transcribed in full. Data were analysed using content analysis to select and organize significant information, to make comparisons between cases and to identify contradictions and outliers. Two researchers independently coded data and discussed this within the multidisciplinary team. This ensured thorough and consistent coding and led to an improvement of the interpretation of coded themes. Data were analysed on an individual (key informant) and group (project) basis. The information was classified according to the changes which have been induced by research results, in particular, impact on informed decision-making, the dissemination of results, and barriers and facilitators to the translation of research. Moreover, the information was classified as expected *versus* achieved use by the levels of the impact CAHS-ROI model. This analysis consisted of classifying some of the significant information according to the different levels of impact and phases of the CAHS-ROI model (Figure 1). The aim was to assess the awareness of the stakeholders involved in the translation of the research results and the differences between expected impacts and those achieved. The results obtained from the expected uses of the projects were then compared with the results of achieved uses, true changes or implementation, as they were described by the informants. In order to compare the utilities detected by the informants in each of the projects as expected or as achieved, their interactions were analysed overlapping the different levels of impact with the phases of the logic model.

Findings were triangulated from different sources (multiple viewpoints: researchers and decision-makers) and perspectives (multidisciplinary researchers and different units of analysis: by project and individual).

## Results

Of the 109 projects funded and completed, six corresponded to research projects on respiratory diseases. Sixteen researchers, both principal and outstanding, from 10 healthcare institutions, were contacted and asked to be interviewed for the study. Of these, 15 agreed to participate; the others did not participate because they could not be reached. When a researcher had participated in more than one project, one interview per project was performed. Hence, the total number of interviews (n = 19) was larger than the number of researchers. As for healthcare decision-makers, 41 people (from seven institutions) were contacted and provided with the questionnaire inquiring about their knowledge of the research projects. Of these, 18 replied, 10 of whom declared being aware of some of the projects on respiratory diseases. Finally, eight healthcare decision-makers (from four institutions) were included in the study sample, the other two were excluded: one because of the impossibility of establishing contact and the other because the poor recording quality of the interview. Baseline characteristics of the projects and the informants are described in Table 1.

**Table 1:** Description of the projects and the key informants

**Project name**	**Period of funding**	**Characteristics of key informants**
P1 - Exacerbation of chronic obstructive pulmonary disease; study of prognostic factors in a cohort of cases	1996-2000	1 PI, 1 I
		2 MD, 1 HS
P2 - Study of the risk factors predisposing to acute exacerbation in patients with chronic obstructive pulmonary disease	1996-1999	1 PI, 5 I
		1 MD, 2 HS
P3 - Validation of a diagnostic procedure in the sleep apnea-hypopnea syndrome, based on the clinical picture and home polysomnography, in the general adult population	1996-1998	1 PI, 2 I
		1 MD
P4 - Cost-effectiveness study of home care in exacerbation episodes of chronic obstructive pulmonary disease by means of the Respiratory-SSIFU (socio-sanitary interdisciplinary functional unit)	1998-2000	3 PI
		5 MD
P5 - Strategies for the management of bacterial resistance in the ICU; application of an empirical antibiotic treatment protocol for ventilation-related pneumonia and impact on the decrease of bacterial resistance and consumption of antibiotics	2000-2003	1 PI
		1 MD
P6 - Phenotypic characterization of chronic obstructive pulmonary disease*	2002-2006	1 PI, 3 I
		2 MD, 2 HS

P: project; PI: Principal investigator; I: Relevant investigator; MD: Hospital medical director; HS: Head of hospital service; *This project continued after the Agency funding ended and therefore only preliminary results were available.

### Changes induced by research results

The impact on decision-making was identified mainly at the intermediate levels of the linear logic model (input-throughout-primary and secondary output-outcome). Most participants indicated changes in health services and/or in clinical practice.

*‘New techniques are hard to incorporate, economically and financially. We had to set up a pneumology day hospital in order to attend to all the patients who were being studied, and we did so. And since we had the day hospital, we took the opportunity to incorporate other services, and this allowed us to continue developing this pneumology day hospital’* (decision-maker, P3).

These changes were mostly indirectly attributed and only in one of the projects the attribution was reported as being direct (P4). Only one of the projects did not result in any achieved impact because at the time of the interview only preliminary results were available (P6). In most of the projects the generation of knowledge or new research has also been identified as direct consequences of the project itself, while in only two (P3 and P5), the economic benefits were stated. Some decision-makers recognized the importance of the projects in contributing to an awareness and sensitivity of managers towards the subject of study (Table 2).

*‘The research project made the hospital management team (manager, general director…) much more sensitive to the problem suffered by patients with apnea cycles’* (decision-maker, P3).

**Table 2:** Description of the changes induced by the research results and their methods of dissemination mentioned by the key informants

**Changes induced by research results**		
	**Classification**	**Examples**
Changes in health services and/or clinical practice	Changes in hospital design and management, such as the creation of a type of service or contribution to the design of chronic care services	*‘After two years… its home-based hospitalization grew as a real service and an integrated care cross-sectional unit was created within the hospital.’* (researcher; P4)
	Changes in staff management	*‘A few years later, that enabled this nurse to become a pneumology case manager nurse. In fact, the only case manager nurse we have in the Consortium is the pneumology nurse.’* (decision-maker; project 3)
	Possible partial contributions to protocols, guidelines and simplifications of procedures; for chronic bronchitis patients, for the treatment and prophylaxis of infections or sleep studies	*‘Yes, indeed, at that time many care protocols were created and in fact with regards to chronic bronchitis patients, the protocols were based at that time on the criteria that we thought was the most adequate in order to prevent as much as possible re-exacerbations.’* (researcher; P1)
	Identification of modifiable risk factors	*‘Yes, some were identified… one of the main results was that physical activity reduced the risk of hospital admission.’* (researcher; P2)
	Contribution to the use of an instrument for the diagnosis of sleep apnea	*‘I can’t guarantee that the validation study has been responsible for this dissemination, but I can say that it was an already validated home process, etc., and it therefore provides some safety, a scientific basis.’* (researcher; P3)
	Change in clinical habits	*‘Yes, it has all generated a culture… We are essentially a clean unit…’* (researcher; P5).
Impact on healthcare costs	In home-based hospitalization	*‘The work supported feasibility arguments… it has an economic impact because it reduces costs, it is safe for the patient, achieves the same results, etc. …’* (researcher; P4)
	Procedure simplification	*‘This study and other studies that we have conducted have proven that the cost of performing one test at the hospital equals the cost of performing at least three others at home…’* (researcher; P3).
Generation of new knowledge	New projects, new studies, new lines	*‘… it generated two or three new related projects… at least three projects separate from the PAC COPD were funded: one on infection, one on inflammation and one on physical activity.’* (researcher; P6)
**Dissemination of knowledge**		
	**Classification**	**Examples**
	Scientific publications	*‘Presentations at scientific meetings by the research team (it consisted of a team in which several hospitals participated), presentations at congresses, and publications, of course.’* (researcher; P2)
	Direct information to potential users	*‘… I think that at the level of clinical sessions … there have been at least three or four sessions that have been important enough to have an impact within the pneumology service, or at least they have had the highest number of patients.’* (researcher; P6)
	Meetings managers - research team	*‘We spoke with the head of the internal medicine service and with the pneumology unit head, which is where the doctor in charge of the project worked, because, in our hospital, these two specialties basically manage the disease.’* (decision-maker)
	Participation and/or collaboration of the decision-maker in the project	*‘… because we participated during the time of elaboration, meaning in the meetings prior to starting the project.’* (decision-maker)
	Proximity of the decision-maker with the research team	*‘I am aware of them because of proximity to the researchers involved, not because of their dissemination.’* (decision-maker)
	Scientific societies	*‘It has been highly disseminated in the European Society of Pneumology …, it has resonated significantly in the European Society of Pneumology, in the American Society of Pneumology, and in the Spanish Society of Pneumology.’* (researcher; P2)
	Clinical Practice Guidelines	*‘… that is included in the COPD clinical practice guidelines, and has led to a change in the behaviour of physicians.’* (researcher; P2)
	Websites	*‘For some time we were uploading results onto a website, within the respiratory section, from the centre.’* (researcher; P2)

### Dissemination of research results

The diffusion of knowledge was mainly done using conventional means, such as scientific publications and clinical practice guidelines, but also in less explicit ways, such as via the involvement of local scientific societies, or in debates and discussions with colleagues and local leaders (P4). The primary channel of this diffusion as expressed by the decision-makers were the meetings held with the research team and/or the heads of healthcare service involved, and this aspect was readily expressed when the managers had collaborated directly in the project (Table 2).

*‘…I am aware of them because of proximity to the researchers involved, not because of their dissemination…’* (decision-maker, P1, P2, P4 & P6).

### Barriers and facilitators

Based on the findings, we made a distinction between internal/external organizational barriers, barriers related to the nature of research, and cultural and individual factors. The organizational barriers were related to the characteristics of the organizational environment and its management (weak coordination between levels of care, frequent rotation of general managers). The nature of research barriers referred to factors related to the characteristics of the type of research itself such as difficulties in the dissemination of results. Finally, cultural and individual barriers were related to the attitudes and beliefs of the researchers (such as resistance to change, or the relationship between the staff who make decisions and the research team). The results are detailed in Table 3.

**Table 3:** Description of barriers (facilitators) mentioned by the key informants

**Classification of barriers**	**Specific barriers**	**Examples**
Organizational barriers/facilitators	Difficulties in relationships between different levels of care, or, in other words, the lack of coordination between them (non-integrated care)	*‘Yes, because we must put primary pharmacologists and hospital specialists on the same wavelength, and so far this task has not been easy. The primary care professional’s perspective doesn’t exactly match the specialist’s; the latter has many more nuances, higher costs and clashes with primary care structures.’* (decision-maker; P4)
	Lack of institutional involvement	*‘It depends on who is involved in the study. If the Catalan Health Service is involved, it should have been willing to offer recommendations that could have subsequently modified or changed clinical practice guidelines…’* (researcher; P4)
	The non-support of clinical managers and planners	*‘The primary care director, the regional management, the primary care division of the Catalan Health Institute and the Foundation for Primary Care Research (Jordi Gol i Gurina Foundation) granted us their support.’* (researcher; P1)
	The frequent changes of managers	*‘Because hospital directors and general managers change frequently, or because this issue is not deemed to be as important as it should be…’* (researcher; P5)
	Lack of channels for the translation of research	*‘The main barrier is that there are no well-established channels for the translation of research. And when researchers finish a study and publish it, there are no other channels, only those created by scientific societies and congresses. This is the main problem. I also think that there are increasing numbers of tools, such as the Health Plan… the Master Plans, which include contact with researchers. More tools like these should be strengthened and developed.’* (researcher; P2)
	Prioritization of research conducted by large groups or centres or the limited opportunities for the promotion of research in primary care centres	*‘I think that healthcare policy is focused on large hospitals and strong research groups, etc., but these clinical aspects of immediate protection, immediate social repercussion, in which the patient is the beneficiary, are somewhat unattended…’* (researcher; P5)
	Previous opportunity in research	*‘Having had the opportunity to receive a first grant, for sure. Having started to think at a good time…’* (researcher; P1)
Barriers/facilitator related with the nature of the research	Difficulties in the dissemination of results due to interdisciplinary reasons.	*‘Clinical journals refused to publish it because they claimed it was too complicated and that clinicians would not understand it… we preach multidisciplinary research but in practice this is more complicated. You can find economists working with clinicians and clinicians working with economists, but a clinical article will rarely be included in an economic journal and vice-versa…’* (researcher, P4).
	Lack of interrelationship between research and industry	*‘First, there have been basic initial research projects with first tests, and then it is crucial that industries have manufactured marketable devices. Therefore, I would say there are two pillars: one, basic research and, the other, the fact that this research has been carried out by industries.’* (researcher; P3)
Barriers/facilitator derived from cultural and individual factors	Reluctance to change	*‘When the idea of change is raised inside a hospital, in the sense of opening up the hospital, having closer ties with primary care, performing interventions outside the hospital, etc., it clashes a bit with the culture of doctors and specialists, but overall when we have carried it out and explained its benefits, we have gotten the approval and participation of many people’* (decision-maker; P4)
	The researcher does not self-attribute the role of disseminating research to managers	*‘Overall, I would say that researchers are forced to take on many roles. I am ready to be a researcher, but not to make an election pamphlet. In this regard, I think the relationship between healthcare policies and health plans is not as it should be.’* (researcher; P6)
	Direct participation of the decision-maker in the project	*‘… because we participated during the time of elaboration, meaning in the meetings prior to starting the project’* (decision-maker; P6 and P2)
	(No) awareness of decision-makers	*‘Directly from the respiratory diseases team I was aware of these projects because I worked in management or in the Drug Commission, and through some of the commissions I was made aware that the project was going ahead…’* (decision-maker; P3 and P1)

### Analysis of expected use *versus* achieved use by levels of impact

**Figure 2:**
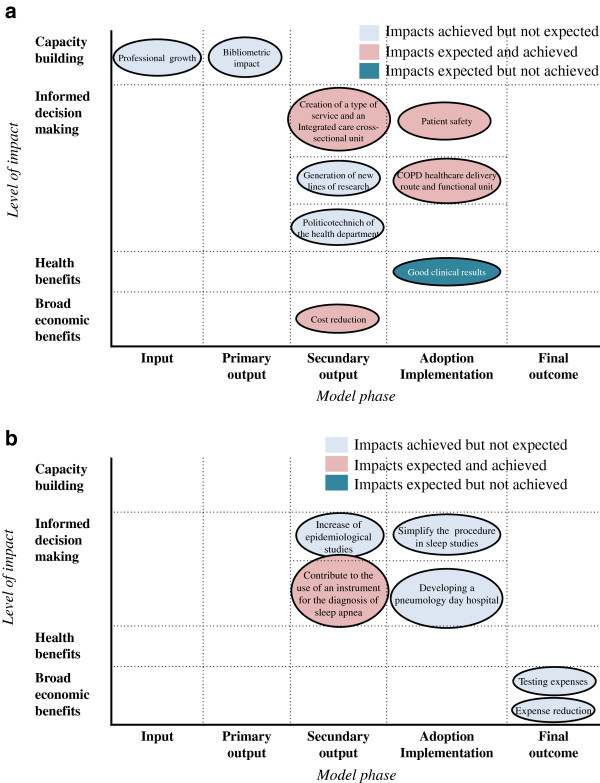
Impacts expected and achieved, by level of impact and model phase in project 4 (a) and project 3 (b).

The broad array of impacts achieved was not expected (unexpected impacts). Also, some expected impacts were not achieved in the last steps of the model (final outcomes and adoption phase). Three projects were considered to have achieved all expected impacts (P1, P3, and P5). Although impacts were mostly reported on informed decision-making, mostly at an intermediate level (secondary outputs such as activity in the field of health or research planning; or in the adoption of results in the healthcare/delivery services phase), other levels of impact were mentioned by the informants. The impact in capacity building was described in all but one project (P6) as an impact that was not expected. Impact in health benefits and broad economic benefits were also achieved in some of the projects (P1, P3, and P4), mainly as final outcomes, while in other cases, these impacts could not be demonstrated yet (P2 and P4). An example of the correspondence between impacts expected and achieved is shown in Figure 2a,b in the cases of project 3 and project 4.

## Discussion

This study, based on a specific group of research projects on respiratory diseases, provides new knowledge on the perspectives of health researchers and decision-makers regarding the decision-making process informed by research findings. Although the study was primarily aimed at analysing impact on informed decision-making, impacts on other categories were also identified and described (capacity building, health benefits, and broad economic benefits). Due to the fact that these levels were not a part of the study objectives, information about them might be less complete.

The findings show that the areas of most frequent impact were those at the intermediate level (secondary outputs). This is consistent with other studies showing that the most frequently recognized impact was that in the areas where the research team had some control and influence. In other words, impacts are typically found in the knowledge production, further research orientation, or in the organizational innovation changes derived from the project [20,21]. For example, regarding achieved impact, nearly all of the researchers reported that their projects have or may have induced changes in clinical practice or healthcare organization: the changes may have been few or many, direct or indirect, and attributable to a greater or lesser extent to the corresponding project. Most of the impacts achieved are beyond the outputs and even the short-term outcomes in the logic model, when the project’s influence would have decreased to become just an indirect influence [22]. The approaches used to transfer new knowledge to clinical and healthcare practice are complex. Local scientific societies and personal connections between researchers and local decision-makers seem to play a very important role. Scientific societies contribute to the diffusion of research results between peers who share a similar context. This takes place usually before a broader diffusion via international conference presentation and scientific publications.

The data obtained have also enabled us to identify specific aspects that may have favoured or hindered the impact of the projects studied. The facilitators/barriers identified are much related to the idiosyncrasy of relationships between the different stakeholders involved. Specifically, teams that include decision-makers or users of health information were more effective in achieving outcomes in health policy or practice from the research findings. The importance of collaboration between researcher and stakeholders has also grown in recognition in other studies [23-25]. This also mirrors one of the conclusions of the study on research impact of the Arthritis Research Campaign, and with one of the recommendations of the CAHS panel that was adopted by Alberta Innovates Health Solutions (formerly the Alberta Heritage Foundation for Medical Research) to promote the inclusion of decision-makers and users in collaborative teams funding programs to promote the transfer of results between the world of researchers and that of management. In other words, the fact that 8 out of the 41 contacted healthcare decision-makers who agreed to be interviewed (prior awareness of at least one of the projects) have ended up having close relationships (often prior to the study) with the research team is probably not coincidental and suggests that it is precisely this relationship or prior joint experience that has facilitated the dissemination of results, their translation and implementation. These decision-makers or users of health information will contribute to or influence the project by conducting research activities; providing expertise, consultation, and clinical advice; lending the project credibility and opening the door for additional sources of funding [26].

Another important facilitator gleaned from the study’s results is the importance of the interrelationship between research and industry (for example, in P3). This factor, raised by the researchers, is very interesting as it reaffirms the pivotal role of industry in product innovation. In another study [27] the biggest predictive factor for the transfer of research to practice and to the market (after the requirements of regulating bodies are met) is the industry’s participation in the earliest phases of research development. Thus, the present study provides definite examples of organizational, cultural, and individual barriers to knowledge transfer (such as negative attitude towards change, unsupportive culture or mutual mistrust) acknowledged in previous reports [25,28] while, at the same time, providing further evidence of the facilitator role that the inclusion of key informants in research projects [28] may have in such transfer processes.

The expected impacts and those achieved enabled gaps between what is expected and what is achieved to be identified (as shown in Figure 2a,b). The classification shows that some achieved impacts were not identified as expected, probably because these were not within the scope of the project, because the interviewees were not aware of the impact itself or because they often confused expected impact with the study’s objective. All this might reflect a low level of engagement between the researchers and the potential users of their research findings. Similarly, the perception of some researchers was that they were not responsible for promoting the application of their results; meaning, the limited awareness of some of the stakeholders involved regarding their role as decision-makers. Moreover, most of the impacts were achieved in areas within the researchers’ control, such as in further research, or in secondary outputs. Similarly, the fact that some expected impacts have not become a reality could simply be a temporal issue given the recent completion of the projects, or as a result of the lack of follow-up studies to identify final outcomes.

The findings of the present study, thus provide some new information on two critical points of impact generation: first, they put researcher expectations within the perspective of actual impact development; and second, they illustrate some examples of *how* knowledge is used, beyond *whether* it is used, which is recognized as a need in impact development research [28].

In fact, our findings may point to the need of expanding the components of the CAHS-ROI model, which has otherwise proved fruitful for our assessment, by illustrating how the interface between research and its results and subsequent feedback may not be linear but rather a complex process depending on several as yet poorly studied components on which action is possible (such as personal communication between professionals or the role of scientific societies). In addition, our findings show how later types of impact beyond knowledge transfer (such as health and economic benefits) may be gleaned from this interview-based study approach as done in other studies [29].

With regards to the limitations of the study, the first, commonly found in this type of studies, consists in the difficulties of specifying the particular and specific impact of the research project studied and the role that other studies may have on new knowledge or organizational changes that took place concurrently. It is unlikely that any of the projects contributed so greatly as to promote definitive changes in clinical and healthcare practice, but most did contribute in some apparently undeniable way to enriching or improving healthcare practice. A second limitation would be the time elapsed between the final period of the funding and the time that this study was done, which could facilitate the achievement of impacts in the older studies. However, we found no big differences between the different projects, probably because they were related to health services research, which is intended to translate knowledge into practice at a much faster rate than other types of research.

Third, the question of whether the conclusions reached are applicable to other projects is raised. It is obvious that this is not the case in a study using a convenience sample, where results can hardly be transferred to other contexts. However, the study aimed to collect points of view (opinions, judgments, values) that may be indicative or descriptive of similar projects and healthcare setting characteristics. On the other hand, some strategies were used to improve validity, such as the triangulation of sources (different viewpoints) or perspectives (units of analyses, consensus interpretation with multidisciplinary researchers) [30]. Moreover, this is an exploratory study and allows for the generation of new results, which should be confirmed by the rest of projects granted.

Finally, knowledge of the interaction between the scientific community and the management community would probably have been better if we had obtained feedback from other types of healthcare decision-makers, given that those who were aware of the projects under study frequently reported having had a relationship with the research teams prior to the projects, often as a result of their similar professional profile. This fact probably facilitated a higher awareness of the results and a greater participation in improvement initiatives that were directly or indirectly related to these results. One could assume that decision-makers who did not respond to the survey probably had fewer relationships with the assessed research projects. This result, in spite of its potential relevance in an overall assessment of healthcare management, was not a study objective. Therefore, there is still a lot to be learnt, and possibly to be done, in this field.

Policy implications might apply in some areas. First, the results proved a number of reasons to advocate for clinical and health services research funding as a means to filling knowledge gaps for improved clinical practice, better healthcare quality, and results. Here one might argue that promoting this type of research (namely T2 type) [16] to be precise, from the public health department or a healthcare organisation that is close to clinical and health services stakeholders (or individuals with close relationship), might increase the chances of impact in improved healthcare services and outcomes. Second, for relevant research targeting one might argue that healthcare decision-makers ought to be included in the research teams. Third, for grant program design and planning in clinical and health services research some ways to improve impacts might be the implementation of policies to encourage knowledge transfer (or exchange) either pushed by the researchers or pulled from decision-makers.

The findings in the present study do not provide specific recommendations for policy makers; rather, they suggest the relevance of researcher/decision maker interaction for implementation policies and thus it makes the case for acknowledging it. As said by other authors [31], assessing and enhancing evaluation culture may help ensure productive research programs: thus, adequate research training and the participation of decision-makers could represent an effective means for influencing the way research is used [32].

## Conclusions

This study provides new knowledge on the perspectives of researchers and healthcare decision-makers regarding informed decision-making based on the results of a specific group of research projects. According to the key informants in this study, the impact of these research projects on decision-making can be direct (application of a finding or an innovation) or indirect, contributing to a complex change in clinical practice or healthcare organization, together with other contextual factors. The channels used to transfer this new knowledge to clinical practice are complex. Local scientific societies and the relationships between researchers and decision-makers can play a very important role. Specifically, relationships between decision-makers and research teams and the mutual knowledge of their activity have been shown to be effective in applying the research. Finally the facilitating factors and barriers identified by the respondents are closely related to the idiosyncrasy of the relationships between the different stakeholders involved.

## Abbreviations

CAHS: Canadian Academy of Health Sciences; ROI: Return of Investment; PIs: Principal investigators; P: Project

## Competing interests

The authors declare that they have no competing interests.

## Authors’ contributions

All authors contributed to the writing of the manuscript. MSD drafted the manuscript, designed the study, and analysed and interpreted the data. PA and IG were involved in designing the study, and analysing and interpreting the data. GPM, JMVP and JE were involved in interpreting the data and revised the manuscript critically for important intellectual content. All authors read and approved the final manuscript.
